# Intraoral fluoride retention after using experimental toothpaste containing titanium tetrafluoride and chitosan

**DOI:** 10.1007/s00784-026-06979-5

**Published:** 2026-06-13

**Authors:** Monique Malta Francese, Julia Pereira Bicalho, Hannah Zomignan Barros, Rafaela Ricci Kim, Gabriela Pellizon Floret, Carolina Ruis Ferrari, Larissa Tercilia Grizzo Thomassian, Deborah Rackel Caldas da Rocha, Ana Carolina Magalhães

**Affiliations:** 1 Department of Biological Sciences, Bauru School of Dentistry, University of São Paulo, Al. Octávio Pinheiro Brisolla, 9-75, Bauru, SP 17012-901 Brazil; 2 Department of Biosciences, Faculty of Dentistry of Piracicaba, University of Campinas, Piracicaba, SP Brazil

**Keywords:** Titanium tetrafluoride, Chitosan, Saliva, Biofilm, Fluoride retention

## Abstract

**Objectives:**

This clinical study evaluated the intraoral fluoride retention using Titanium tetrafluoride (TiF_4_)/Chitosan (Ch) toothpaste.

**Materials and methods:**

Participants (*n* = 20) were randomly divided into double-blind and crossover phases according to the toothpastes: (1) TiF₄/Ch (1400 ppm F^−^, pH 4.5, 0.5% chitosan); (2) TiF₄ (1400 ppm F^−^, pH 4.5); (3) Elmex^®^ Caries Protection (positive control, 1450 ppm F^−^, AmF, pH 4.5); and (4) Placebo (negative control, pH 7.0). Each participant received all treatments, with a 7-day washout period between phases. After seven days, stimulated saliva and biofilm samples were collected 1 h and 12 h after brushing. Fluoride concentrations (ppm F^−^) were determined with specific electrodes and statistically analyzed (ANOVA/Tukey, *p* < 0.05).

**Results:**

After 1 h, TiF₄/Ch toothpaste increased the mean fluoride levels in saliva (4.33 vs. 0.81 ppm for the placebo), biofilm fluid (0.25 vs. 0.18 ppm), solid biofilm (12.66 vs. 3.73 ppm), and total biofilm (22.14 vs. 3.19 ppm), similarly to TiF₄ and Elmex^®^. After 12 h, mean fluoride levels in saliva and biofilm fluid returned to baseline, while elevated levels persisted in the solid biofilm (8.29 vs. 3.15 ppm) and total biofilm (6.57 vs. 2.03 ppm).

**Conclusions:**

TiF₄/Ch toothpaste maintained fluoride retention in stimulated saliva and biofilm, similarly to the TiF_4_ and Elmex^®^. Fluoride retention in saliva and biofilm fluid was observed at 1 h after use, whereas in biofilm it persisted for 12 h.

**Clinical relevance:**

TiF₄/Ch toothpaste showed prolonged fluoride retention in biofilm, comparable to the commercial toothpaste, supporting its potential as an alternative for maintaining intraoral fluoride reservoirs.

**Trial registration:**

This study was registered at the Brazilian Registry of Clinical Trials (ReBEC) under number RBR-3f5c5hx.

## Introduction

“Dental caries is a biofilm-mediated, diet-modulated, multifactorial, non-communicable and dynamic disease resulting in net mineral loss of dental hard tissues. It is determined by biological, behavioral, psychosocial, and environmental factors” [1]. Cariogenic microorganisms can metabolize different types of sugars from the diet, producing acids that change the biofilm pH values below 5.5 and cause demineralization of dental enamel [1–3]. When this process occurs frequently, it disrupts the equilibrium between demineralization and remineralization, and carious lesion can develop as a consequence of this process [1].

In addition to reducing sugar consumption, another important strategy for controlling dental caries is the application of fluorides. The use of fluoride is considered a significant advancement in public health [4], due to the reduction in the incidence and severity of dental caries lesions found in clinical trials [5–7]. In special, fluoridated toothpastes are important for daily biofilm disorganization by brushing, to prevent the permanence of cariogenic microorganisms and, consequently, the development of carious lesions [8]. Its benefit is primarily due to its capacity to increase the intraoral fluoride (F) reservoir to levels high enough to affect the balance between enamel demineralization and remineralization overtime [9].

Oral mucosa has been highlighted as an important reservoir of fluoride due to its ability to release this component into saliva over time [9]. During toothbrushing, fluoride from the toothpaste is dispersed through the toothpaste-saliva slurry, allowing direct contact with the tooth surface and residual biofilm. After brushing, part of the fluoride may remain temporarily retained in oral soft tissues and be gradually released back into saliva, contributing to fluoride availability in the oral environment over time. Because biofilm is constantly exposed to saliva, both the immediate fluoride availability during brushing and its subsequent release from oral reservoirs may influence fluoride levels in this compartment [9]. Since teeth are constantly exposed to saliva, the constituents, chemical characteristics and properties of this oral fluid play a crucial role in the occurrence and progression of caries lesions [10]. However, from a clinical perspective, intraoral fluoride retained in the biofilm is likely more relevant, since it has direct contact with enamel [9].

Sodium Fluoride (NaF) and amine fluoride (AmF) are well established in the literature as conventional F anti-caries agents and they are present in most of the commercial products for home care and professional application. Their anticaries effect in toothpastes is mainly related to the incorporation of fluoride into the residual dental biofilm not removed by brushing, together with the temporary retention of fluoride in oral soft tissues, from which it can be subsequently released back into saliva. These intraoral reservoirs contribute to maintaining fluoride availability in the biofilm fluid after toothbrushing, which may help reduce enamel demineralization [9]. The precipitation of calcium fluoride (CaF₂) on enamel occurs only in small amounts and plays a minor role under these conditions [11–13]. In patients with high sugar consumption, however, these conventional fluorides may be insufficient, and additional measures may be required.

Based on this, recent studies have focused on fluoride compounds that may provide additional protective effects, such as those fluoride containing polyvalent metals, through surface precipitation or incorporation of ions into demineralized tissue, especially Titanium Tetrafluoride (TiF₄) [12]. The potential superiority of TiF₄ over conventional fluorides has been attributed not only to fluoride availability, but also to the action of titanium, which seems to interact with the phosphate from tooth apatite and form a glaze-like layer on the tooth surface rich in titanium oxide and hydrated titanium phosphate, reported to be more acid-resistant than the CaF₂-like layer [12]. In addition, due to its low pH, TiF₄ may induce higher fluoride deposition on the clean tooth surface compared with NaF [12]. Studies have shown promising results for TiF_4_ based products in preventing tooth demineralization, in both dental caries and erosion conditions [14–31]. In some cases, TiF_4_ varnishes and solutions products have demonstrated even greater efficacy compared to conventional fluorides, under models simulating dental caries in vitro and clinically [17, 22, 25].

Our research group has evaluated an experimental toothpaste containing TiF₄ and chitosan (Ch), demonstrating its potential to minimize erosive tooth wear in vitro and in situ [28, 30]. The addition of chitosan in TiF_4_ toothpaste is justified once this biopolymer can adsorb onto the enamel, creating a positively charged and more hydrophobic surface, which serves as a mechanical barrier against acids, preventing their penetration and contributing to the prevention of demineralization [23, 32].

Despite some evidence exist about the protective effect of TiF_4_ toothpaste against tooth demineralization, no data are available about the retention of F in biofilm and saliva after its use. Therefore, this study played a crucial role in elucidating the mechanism of action involved in the effect of this experimental toothpaste, as well as its influence on the oral cavity over time, considering its potential future commercialization. Therefore, the study aims to evaluate an experimental toothpaste containing Titanium Tetrafluoride (TiF_4_)/Chitosan (Ch) in terms of intraoral fluoride levels in saliva, biofilm fluid, solid biofilm and total biofilm compared to a conventional fluoride containing toothpaste in vivo.

## Materials and methods

### Ethical considerations and participants

The study was approved by the Ethics Committee of Bauru School of Dentistry – University of São Paulo (FOB/USP, CAAE n° 77660824.5.00000.5417) and was conducted in accordance with the guidelines of the institutional research committees, and with the Declaration of Helsinki. All adult participants received and signed a written informed consent form prior to their inclusion in the study. Participant confidentiality was ensured through anonymized data handling. The reporting of this randomized clinical trial followed the CONSORT guidelines. The trial was registered at the Brazilian Registry of Clinical Trials (ReBEC) under number RBR-3f5c5hx.

Twenty participants (4 men and 16 women, aged 18–31 years) were selected in the study after reading the Letter of Clarification to the Research Participant and signing the Free and Informed Consent Form. The number of participants was predetermined based on previous studies that employed protocols similar to the present work [33, 34]. A small pilot assessment with two participants was also conducted to verify the feasibility of the clinical procedures, sample collection and laboratory analyses.

General health was assessed using an online form (Google Forms^®^) previously made available to participants who expressed interest in participating in the study, aiming to collect preliminary data and initial selection. Oral health was assessed through a clinical examination.

The inclusion criteria required that selected participants were residents of the same city (Bauru, São Paulo State, Brazil; 0.77–0.87 mg F/L in the public water supply network), had good general and oral health (no active or cavitied caries lesions or signs of gingivitis/periodontitis), a stimulated salivary flow (SF) rate > 1 mL/minute and an unstimulated salivary flow (UNF) rate > 0.25 mL/minute. The exclusion criteria included the presence of systemic disease, pregnancy or breastfeeding, use of orthodontic appliances, professional application of fluoride 2 months prior to the study, and smoking or medications use that could affect salivary flow rate and/or biofilm formation [30, 33].

The study posed minimal risk, limited to transient discomfort related to routine dental procedures and saliva collection. Participation was voluntary, with no guaranteed direct clinical benefit, although participants may have indirectly benefited from oral examination, professional prophylaxis, and contribution to scientific knowledge. Participants did not receive any financial compensation for participation. They received a final professional cleaning, as well as breakfast after each collection day.

### Preparation of toothpastes

To ensure the quality control of the experimental toothpastes, the formulations were prepared internally in the biochemistry laboratory using components sourced from Bauru Formulas pharmacy (Bauru, SP).

The tested formulations are detailed in Table 1. The base formulation was prepared by first mixing carboxymethylcellulose, glycerin, and a portion of warm water, in this specific order, to obtain the appropriate consistency. The remaining excipients included methylparaben, sorbitol, abrasive silica, titanium dioxide, cocamidopropyl betaine, mint flavoring, and water q.s. The placebo had the same base formulation, but without fluoride or chitosan. After preparation, the toothpastes were filled into identical coded white tubes and stored under refrigerated conditions. The formulations were prepared considering an estimated shelf life of approximately 3 months, according to standard practice in compounding pharmacies; however, no specific stability tests were performed to determine whether this period could be extended. The pH of the toothpastes was measured in duplicate using a previously calibrated pH meter (Orion 3-star pH Bench Top, Thermo Electron Corporation, USA) with pH 4.1 and 7.0 standards [28]. The pH of all experimental toothpastes, except for the placebo, was adjusted to 4.5 using 0.1 M sodium hydroxide (NaOH) solution, corresponding to the commercial pH value of the toothpastes (Table 1). The placebo toothpaste retained its native neutral pH.

**Table 1:** Composition of the toothpastes under study and their respective F concentration and pH values

Toothpaste	Manufacturer	Base and active components, respectively		Fluoride content	pH
TiF_4_/Ch	Laboratory - FOB/USP	carboxymethylcellulose (CMC), glycerin, methylparaben, sorbitol, abrasive silica, mint flavoring, titanium dioxide, cocamidopropyl betaine, water q.s.	TiF_4_ (Sigma-Aldrich^®^, 333239) and 0.5% chitosan (75% deacetylation, 500 mPas, Chitoscience^®^, 23306)	1400 ppm F^-^	4.5
TiF_4_	Laboratory - FOB/USP	carboxymethylcellulose (CMC), glycerin, methylparaben, sorbitol, abrasive silica, mint flavoring, titanium dioxide, cocamidopropyl betaine, water q.s.	TiF_4_	1400 ppm F^-^	4.5
Elmex^®^ Caries Protection	GABA International AG, Grabetsmattweg, Switzerland	glycerin, sorbitol, hydrated silica, flavor, cocamidopropyl betaine, sodium saccharin, hydroxyethylcellulose, water	amine fluoride (AmF)	1450 ppm F^-^	4.5
Placebo	Laboratory - FOB/USP	carboxymethylcellulose (CMC), glycerin, methylparaben, sorbitol, abrasive silica, mint flavoring, titanium dioxide, cocamidopropyl betaine, water q.s.	none	none	7.0

Chitosan was filtered through filter paper to enhance its integration with the other components of the experimental toothpastes. The toothpastes containing Ch were initially prepared by dissolving 1% of the respective type of chitosan in a 1% acetic acid solution, which was then mixed with water in a 1:1 ratio [23, 24, 28].

Total Fluoride (TF), Soluble Fluoride (SF), and Ionic Fluoride (IF) present in the toothpastes were quantified as previously described, to assure at least 1000 ppm F^−^ available [14]. A single aliquot from each toothpaste batch used in the in vivo protocol was analyzed in duplicate.

### In vivo study design

**Fig. 1:**
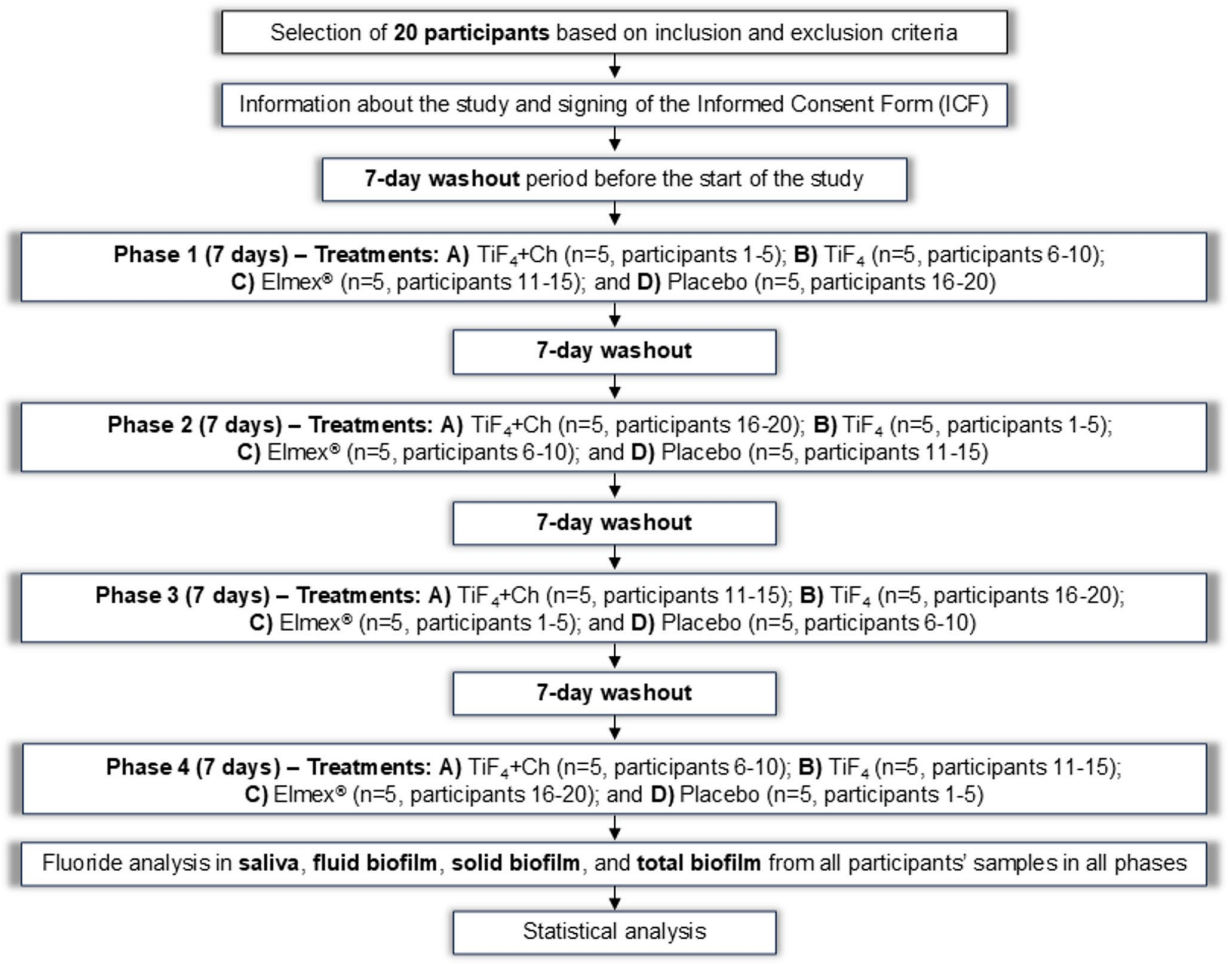
Study flowchart

This study had an in vivo double-blind and crossover design (Fig. 1). Both the participants and the researchers responsible for sample collection and clinical procedures were unaware of the toothpaste allocation throughout all experimental phases (Fig. 1). Participants were randomly assigned to four possible treatments: (1) Experimental toothpaste containing TiF_4_ (1400 ppm F^−^)/Ch at 0.5% (75% deacetylation, 500 mPas, pH 4.5); (2) Experimental toothpaste containing TiF_4_ (1400 ppm F^−^, pH 4.5); (3) Commercial toothpaste Elmex^®^ caries protection (GABA, Switzerland, 1450 ppm F^−^, AmF, positive control for caries, pH 4.5); and (4) Placebo toothpaste without F (negative control, pH 7.0). The participants had no prior knowledge about the toothpastes tested during the experiment. All toothpastes were inserted into identical opaque white tubes, and each tube was labeled only with a color code determined by an independent researcher who was not involved in participant contact, clinical procedures, or outcome assessment (A.C.M). The random allocation sequence was generated using Microsoft Excel^®^ (in balanced blocks) and implemented through coded toothpaste tubes.

The participants were instructed to keep their usual eating habits and to perform oral hygiene using the materials provided by the researchers to each participant. A washout kit, consisting of a toothbrush (Curaprox^®^, Kriens, Switzerland), dental floss (Needs^®^, Brazil), a Placebo toothpaste (without F) and a cup for post-brushing rinsing, was provided before the first phase and between the 4 treatment phases. At the beginning of each experimental phase, a new treatment kit containing the same items was provided (changing the toothpaste). After each phase, an acceptability form (Google Forms^®^) was applied to assess the participants’ degree of satisfaction with the flavor and texture of treatment (scores from 0-worst to 10-best) and difficulties during each treatment phase.

The participants were also instructed to refrain from using any other type of fluoride or antimicrobial product during the study. Before the beginning of the study, participants attended an in-person orientation session, during which detailed instructions were provided and the brushing procedure was verified by the researchers. During the experiment, the WhatsApp group used for communication was restricted so that only the administrators could send messages. Compliance with dietary and oral hygiene instructions was reinforced verbally and in written form at the beginning of each experimental phase, and participants were followed through instant messaging groups to clarify instructions and to reinforce adherence throughout the study period.

Before the start of protocol, the participants were submitted to dental prophylaxis to remove all accessible biofilm and dental calculus. Participants were instructed to brush their teeth 3x/day - standard practice among adults in Brazil [33, 34] using approximately 1 g of toothpaste per brushing session for 1 min, and 10 mL of running water to rinse their mouth after brushing. Each experimental phase lasted 7 days [34]. On day 6 of the study, participants were instructed to refrain from brushing and flossing their teeth. Instead, they were allowed to brush only the occlusal surface, to allow biofilm to accumulate on the smooth surface. After going to bed, they were instructed not to consume food or liquids, except water, until the next day (day of the saliva and biofilm collection) [33, 34]. Therefore, on the collection day, no food intake was allowed before sample collection.

In the morning after seven days of thrice-daily use of the toothpastes, the first dental biofilm samples were collected (right side of the mouth, 12 h of the last brushing). Subjects then brushed the occlusal surface according to their respective treatment group for 1 min and rinsed their mouth with 10 mL of tap water for 5 s. Dental biofilm samples were collected again 1 h later (left side of the mouth, 1 h of brushing). The entire protocol was repeated for the other three toothpastes not previously used and, between each phase, a washout period of 7 days was allowed in order to minimize any residual effect.

Dental biofilm was collected from all accessible buccal and lingual surfaces using sterile curettes (Quinelato^®^, n^o^. 11/12, Rio Claro, Brazil). The sampling area was divided according to the dental arch: upper teeth (molars only) were used for biofilm fluid and solid biofilm analyses, and lower teeth (all teeth) were used for total biofilm analysis. This site-specific approach was adopted for methodological reasons: upper molars provided greater biofilm accumulation, allowing separation of the fluid and solid fractions after centrifugation, whereas in lower teeth this separation was not feasible, and the collected material was therefore used for total biofilm analysis.

Biofilm samples were immediately transferred into tubes filled with mineral oil (Silicones Paulista Ind. Com. de Prod. Quím. Ltda., São Paulo, SP, Brazil). After determining the wet sample weight (± 10 µg), half of the tubes were centrifuged for 10 min (21,000 g, 4 °C) to separate the fluid from the biofilm solids (upper teeth). The biofilm fluid was then collected using glass capillary micropipettes, kept between two small aliquots of mineral oil, which is less dense than water. The purpose of the mineral oil was to prevent biofilm fluid evaporation. The glass capillary micropipettes were manufactured in the Faculty of Dentistry of Piracicaba (FOP) laboratory using glass capillaries that were inserted into a glass microelectrode extractor (Narishige, Japan, Model PP-830).

Then, the other half of the biofilm tubes were directly stored to compose the total biofilm (lower teeth), without centrifugation, in order to preserve the entire collected material for subsequent analysis. All biofilm samples (fluid, solid, and total) were kept frozen until the end of the in vivo protocol.

Stimulated saliva was collected for 5 min (1 and 12 h after the last use of the toothpastes), centrifuged (12,000 rpm, 5 min), and the supernatant was collected for subsequent fluoride analysis. Saliva samples were also kept frozen until the end of the in vivo protocol.

Fluoride analysis of the samples followed different protocols due to the specific characteristics of the fluid microsamples, as described below. Stimulated saliva, total biofilm, and solid biofilm were analyzed using the HMDS-facilitated diffusion method, whereas biofilm fluid was analyzed using the inverted fluoride electrode method, which is suitable for nanolitre-scale samples.

### Fluoride analysis in saliva

For fluoride analysis in stimulated saliva samples, petri dishes were perforated at the edge of the lid and labeled. A 1 g of sample was weighed twice onto the plates to perform duplicate analysis. The uncovered plates were distributed on the bench and petroleum jelly was applied to the edge of the petri dish lid. The volume of deionized water necessary to complete a total of 2 mL was pipetted, considering the individual weight of each sample. On the inner side of the lid, opposite the hole, 50 µL of 0.05 M NaOH were pipetted in 5 drops (10 µL per drop). The plates were closed with the aid of petroleum jelly applied to the lid. Then, 2 mL of hexamethyldisiloxane (HDMS) were added through the hole in the lid, which was immediately sealed with petroleum jelly and a cutout of Parafilm. The samples remained under agitation for at least 3 h for analysis. After 3 h, the plates were opened and the contents at the bottom of the plate were discarded, assuming that all the fluoride in the sample had been transferred to the 5 drops of sodium hydroxide (NaOH). On the lid, 25 µL of 0.2 M acetic acid was pipetted and added to the 5 drops of NaOH; the entire content was aspirated with a pipette, completing the final volume to 75 µL of deionized water. The resulting droplet was taken to the ion-specific electrode (model Orion 9409BN) coupled to a potentiometer (Procyon, model SA 720) for measurement, and the data were tabulated in Microsoft Excel^®^ with mV-to-ppm conversion performed using a calibration curve. This curve (0.25 to 10 nmol F) was prepared in triplicate from a fluoride standard solution (NaF, 100 ppm, Orion nº 940907), and the potentials (mV) obtained for each standard were plotted against the log of fluoride concentration (r² ≥ 0.99).

### Fluoride analysis in total biofilm and solid biofilm

For fluoride analysis in total and solid biofilm samples, the protocol was similar to the saliva analysis; however, a duplicate analysis could not be performed due to the low sample quantity of biofilm. Before the analyses, the mineral oil was carefully aspirated with a glass capillary micropipette and discarded (only for the solid biofilm samples). The samples were placed into microtubes, which were then incubated in an oven at 80 °C for 2 h to determine the dry weight of biofilm and then, submitted to the F analysis protocol as described above. The data were tabulated and converted as previously described for the saliva samples.

### Fluoride analysis in biofilm fluid

Determination of the fluoride concentration in Biofilm Fluid was carried out using the inverted fluoride electrode method [35, 36], which permits analysis of nanolitre volumes of samples. The capillary micropipettes were transported to the FOP laboratory for analysis. The lubricating oil present at the tip of the capillary micropipette was discarded, and the fluid was deposited in the form of “droplets”, maintaining a consistent size ratio on the crystal of the fluoride ion-specific electrode (model Orion 9409BN - adapted), which was adapted for microanalysis and previously calibrated using fluoride standards (1.0–1,000 µM F). These droplets were approximately one-tenth of the volume of the standard drops (1:10 TISAB III). To measure each sample, the reference electrode was inserted into the droplet on the crystal. The data obtained using the specific software were subsequently transferred to a Microsoft Excel^®^ spreadsheet for backup. Due to technical limitations in collecting biofilm fluid, fluoride analysis was performed only in samples with sufficient volume (*n* = 10). The inverted fluoride electrode method allows highly sensitive microscale analysis.

### Statistical analysis

The fluoride concentrations in saliva, biofilm fluid, solid biofilm, and total biofilm were tabulated in Microsoft Excel^®^ spreadsheets. GraphPad software (San Diego, USA) was used to perform the statistical analysis. All randomized participants (*n* = 20) completed the four experimental phases and were included in the final analysis (per-protocol). We assessed whether the data were normally distributed and homogeneous using the Kolmogorov-Smirnov test and Bartlett’s test, respectively. After this verification, the most appropriate statistical test for the results was applied (one or two 2-way ANOVA/Tukey or Kruskal-Wallis/Dunn). The significance level adopted for all tests was set at 5%.

## Results

### Analysis of total, soluble and ionic fluorides in toothpastes

The results of the analysis of ionic, soluble, and total fluoride concentrations in the different toothpastes revealed variations between the formulations. All toothpastes, except for the placebo, exhibited fluoride concentrations above 1000 ppm F^−^ (Table 2).

**Table 2:** Mean ± standard deviation of total, soluble, and ionic fluoride (ppm F^−^, µg F/g) in the toothpastes

Toothpaste	Ionic Fluoride (IF)	Total Soluble Fluoride (TSF)	Total Fluoride (TF)
TiF_4_/Ch	1097.5 ± 9.1	1093.2 ± 2.1	1028.8 ± 5.7
TiF_4_	1168.6 ± 16.2	1159.4 ± 2.3	1283.9 ± 17.8
Elmex^®^	1271.3 ± 7.0	1249.1 ± 7.3	1222.5 ± 6.8
Placebo	16.2 ± 0.4	14.0 ± 0.1	14.6 ± 0.0

^a^ Values obtained from a single aliquot of each toothpaste batch used in the clinical protocol, analyzed in duplicate

### Fluoride analysis of saliva

Table 3 shows the fluoride concentrations in saliva. Fluoridated toothpastes resulted in a significant increase in fluoride levels 1 h after use compared to the placebo, with no significant differences among them. After 12 h, no differences were observed between treatments; however, a significant reduction in fluoride concentrations in saliva was observed for all fluoridated treatments, except for the placebo.

**Table 3:** Fluoride concentrations (mean ± SD) in saliva (ppm F^−^) and fluid biofilm at 1 h and 12 h after brushing with different toothpastes

	1 h	12 h
	**Saliva**	
TiF_4_/Ch	4.33 ± 3.55 Aa	1.06 ± 1.02 Ab
TiF_4_	3.41 ± 3.21 Aa	1.07 ± 1.11 Ab
Elmex^®^	3.72 ± 4.22 Aa	0.90 ± 0.65 Ab
Placebo	0.81 ± 0.70 Ba	0.86 ± 0.62 Aa
	**Fluid biofilm**	
TiF_4_/Ch	0.25 ± 0.06 Aa	0.20 ± 0.05 Aa
TiF_4_	0.24 ± 0.06 Aa	0.20 ± 0.06 Aa
Elmex^®^	0.26 ± 0.06 Aa	0.19 ± 0.06 Aa
Placebo	0.18 ± 0.06 Ba	0.21 ± 0.07 Aa

^a^ Fluoride concentrations in saliva (*n* = 20): two-way analysis of variance (ANOVA) (treatment: *p* = 0.0018; time: *p* < 0.0001; interaction: *p* = 0.0019). It was followed by Tukey’s post hoc test

^b^ Fluoride concentration in fluid biofilm (*n* = 10): one-way analysis of variance (ANOVA) (1 h: *p* = 0.0084 for treatments; 12 h: *p* = 0.7958 for treatments). It was followed by Tukey’s post hoc test

^c^ To compare the time points (1 h vs. 12 h) for fluid biofilm, within each treatment group, a t-test was applied (Elmex: *p* = 0.0585; Placebo: *p* = 0.4202; TiF₄: *p* = 0.1572; TiF₄ + Ch: *p* = 0.1277)

^d^ Different uppercase letters indicate significant differences between treatments at each time point. Different lowercase letters indicate significant differences between 1-hour and 12-hour measurements within each treatment group

### Fluoride analysis of fluid biofilm samples

Table 3 also shows the fluoride concentrations in the biofilm fluid. All fluoridated toothpastes resulted in a significant increase in fluoride levels 1 h after use compared to placebo, but with no difference between them. After 12 h, no differences were observed between the toothpastes with respect to the fluoride concentrations in biofilm fluid. Furthermore, no differences were found between 1 and 12 h in all treatment groups.

### Fluoride analysis of solid and total biofilm samples

Table 4 shows the fluoride concentrations in the total and solid biofilm at different times after the use of the tested toothpastes. After 1 h, the fluoridated toothpastes showed significantly higher concentrations than the placebo. Even after 12 h, these concentrations remained high in the fluoridated toothpastes, while the placebo consistently maintained low levels at both times. No significant differences were observed among the fluoridated toothpastes in both periods. There was a reduction in F level for fluoridated toothpastes overtime, comparing 1 and 12 h.

**Table 4:** Fluoride concentrations (mean ± SD) in solid and total biofilm (ppm F^−^) at 1 and 12 h after brushing with different toothpastes

	1 h	12 h
	**Solid biofilm**	
TiF_4_/Ch	12.66 ± 9.32 Aa	8.29 ± 6.48 Ab
TiF_4_	12.66 ± 8.97 Aa	7.41 ± 5.09 Ab
Elmex^®^	20.13 ± 15.98 Aa	12.47 ± 8.68 Ab
Placebo	3.73 ± 2.53 Ba	3.15 ± 1.25 Ba
	**Total biofilm**	
TiF_4_/Ch	22.14 ± 10.77 Aa	6.57 ± 4.78 Ab
TiF_4_	23.06 ± 10.85 Aa	6.06 ± 2.96 Ab
Elmex^®^	21.80 ± 10.14 Aa	11.32 ± 6.98 Ab
Placebo	3.19 ± 2.31 Ba	2.03 ± 1.83 Ba

^a^ Fluoride concentrations in solid biofilm (*n* = 20): two-way analysis of variance (ANOVA) (treatment: *p* < 0.0004; time: *p* = 0.0004; interaction: *p* = 0.1221)

^b^ Fluoride concentration in Total biofilm (*n* = 20): two-way analysis of variance (ANOVA) (treatment: *p* < 0.0001; time: *p* < 0.0001; interaction: *p* < 0.0001)

^c^ Both analyses were followed by Tukey’s post hoc test

^d^ Different uppercase letters indicate significant differences between treatments at each time point. Different lowercase letters indicate significant differences between 1-hour and 12-hour measurements within each treatment group

### Acceptability of the toothpastes

**Fig. 2:**
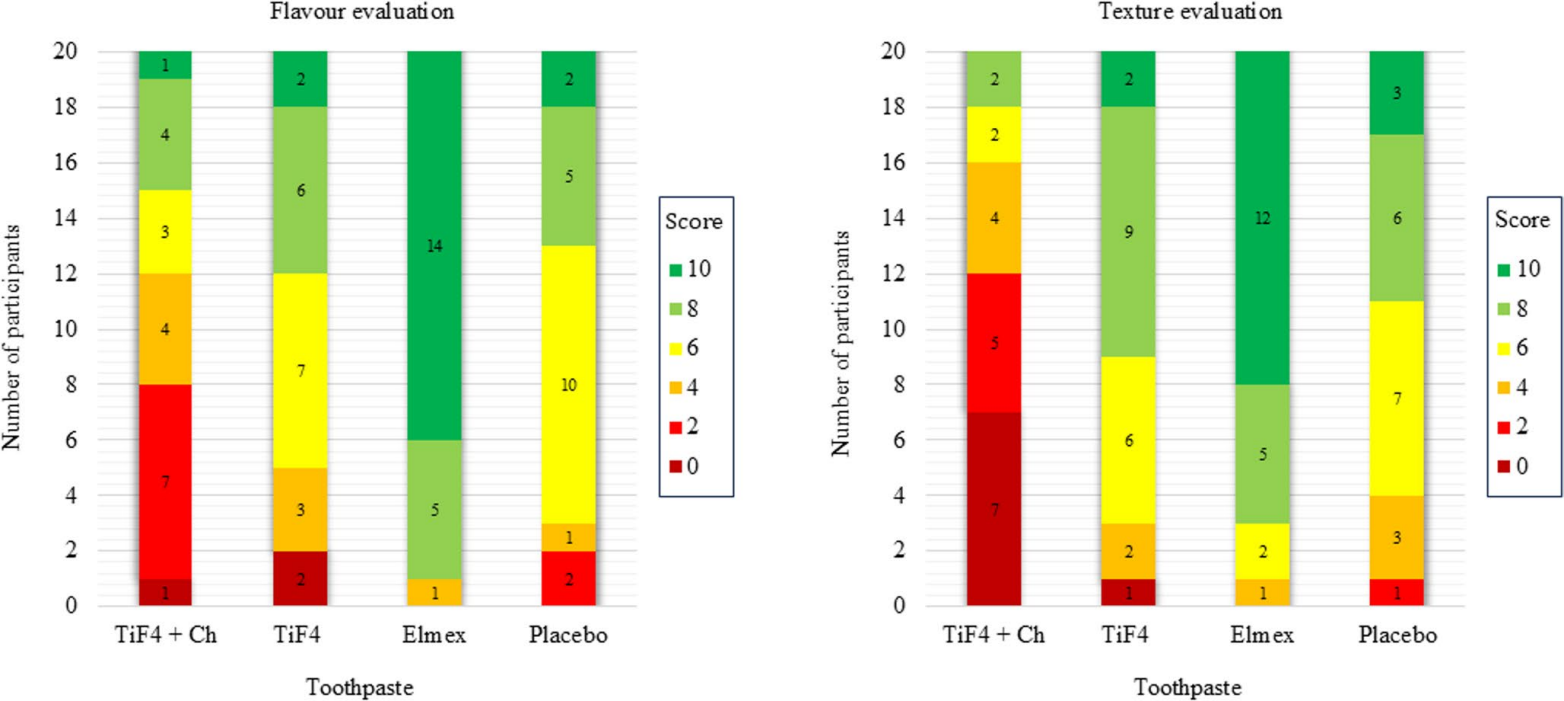
Distribution of participants’ scores (0–10) for flavor and texture evaluation of the tested toothpastes

Figure 2 shows the acceptability of the toothpastes by the participants (flavor and texture). This evaluation was conducted using independent questionnaires applied at the end of each treatment phase (4 phases, score from 0 to 10). The Elmex^®^ caries protection toothpaste was the product that pleased the participants the most in terms of flavor and texture, followed by the placebo. The toothpaste that was least pleasing in terms of flavor was the TiF_4_/Ch. Tooth yellowing was also reported by participants in response to the experimental toothpastes (35% of participants for TiF_4_/Ch, 50% for TiF_4_, and 15% for placebo), whereas no reports were associated with the commercial toothpaste (Elmex^®^: 0%).

## Discussion

To our knowledge, no published studies have evaluated intraoral fluoride retention after the use of TiF₄ toothpastes. In the present study, the fluoridated toothpastes increased fluoride levels after 1 h in saliva and the biofilm compartments compared with placebo, while higher fluoride levels persisted after 12 h in solid and total biofilm. No significant differences were observed among the fluoridated toothpastes. Considering that these sites are in direct contact with the tooth surface, the analysis of fluoride concentrations is essential from a clinical perspective, as fluoride retention in oral reservoirs interferes with the demineralization and remineralization processes of dental hard tissues, particularly in the biofilm fluid, where chemical interactions take place during the development of dental caries [9].

It is important to highlight that the presence of Chitosan in the TiF₄ formulation did not influence fluoride release, as similar fluoride concentrations were observed in both TiF₄ and TiF₄/Ch toothpastes. These findings suggest that, under the conditions tested, chitosan did not alter fluoride availability in the oral environment. Furthermore, the presence of titanium (a polyvalent metal) also had no influence on F release and retention, once no differences were found between toothpastes containing TiF_4_ and AmF.

Although the TiF₄ toothpaste was not superior to the commercial Elmex^®^ Caries Protection toothpaste in terms of intraoral fluoride retention, its comparable performance remains relevant. Previous studies have shown that formulations with TiF₄ may be effective in minimizing erosive tooth wear in enamel and dentin under in vitro and in situ conditions [28, 30], and have also demonstrated promising in vitro results for enamel and dentin caries control [26, 27], in both cases compared to conventional fluorides. Therefore, the present findings support further investigation on the effect of this toothpaste formulation in biofilm models to reduce tooth demineralization, without allowing, at this stage, direct extrapolation of an anticaries effect from the current intraoral data. If future studies confirm its potential for dental caries, this formulation may represent an interesting alternative as a single product with broader preventive applicability. Although no additional benefit of chitosan was observed under the conditions tested, its inclusion cannot yet be definitively ruled out, since further investigation in more specific caries-related protocols is still needed before drawing a final conclusion about its contribution to TiF₄ toothpaste efficacy.

Studies have shown that, after the administration of fluoridated formulations, fluoride concentration in saliva increases immediately, reaching high levels within the first hour. However, these levels decrease drastically over time and return to values close to baseline within 3 to 6 h [33, 34, 37–39], which is consistent with the results of saliva and biofilm fluid analysis of the present study. The greater persistence of fluoride in biofilm fluid reported by some studies [40–42] may be related to methodological differences. Frequent cariogenic challenges (absent in our protocol), such as repeated sucrose exposures, could favor fluoride release from solid biofilm into the fluid phase and explain the results of previous reports [40–42].

Thus, the present study suggests a short-term increase in fluoride levels in saliva and biofilm fluid after toothbrushing, while persistent fluoride retention was evident in the solid and total biofilm fractions. A statistical power analysis showed that the comparison between TiF_4_/Ch and placebo achieved statistical power greater than 80% for saliva at 1 h, solid biofilm at 1 and 12 h, and total biofilm at 1 and 12 h (92.4% to 100.0%). However, the interpretation of saliva at 12 h and biofilm fluid, particularly at 12 h, should be made with caution because these comparisons presented lower statistical power and, in the case of biofilm fluid, were based on a reduced number of available samples (*n* = 10). Moreover, because no intermediate time point between 1 and 12 h after brushing was evaluated, the kinetics of fluoride retention and clearance within this interval could not be determined.

Similarly to our study, Kondo et al. [34] observed that 1 h after brushing, the use of a conventional toothpaste containing 1100 ppm F^−^ increased fluoride concentrations in saliva and biofilm fluid. After 12 h, concentrations in both compartments returned to baseline, indicating that fluoride retention in saliva and biofilm fluid is predominantly transient and reflects a short-lived intraoral reservoir. In the present study, however, salivary fluoride levels were higher than those found in biofilm fluid, possibly because part of the fluoride in biofilm was associated with the solid fraction [36]. Methodological differences may account for discrepancies in absolute values between studies, since Kondo et al. [34] analyzed all samples, including saliva, using an oil-covered inverted fluoride electrode, whereas in the present study this technique was applied exclusively to biofilm fluid samples.

On the other hand, studies have shown that fluoride concentration remained significantly higher even over longer periods in biofilm [34, 37, 39, 43], which was also in agreement with the results of total and solid biofilm found in the present study. Differently from our results, Pessan et al. [33] could not find significant difference between the 1000 ppm F^−^ toothpaste and the placebo after 12 h in total biofilm (30.1 and 14.2 ppm F^−^, respectively). This may be related to the shorter brushing period used in Pessan’s study (4 days) compared to the present study (7 days). Furthermore, the formulation of the toothpastes was not disclosed, and excipients could have influenced fluoride release and retention. In the present study, a toothpaste containing 1450 ppm F^−^ as AmF was used instead of 1000 ppm F^−^ as NaF tested by Pessan et al. [33], which could have contributed to the observed differences. In fact, the absolute values of fluoride in the total biofilm observed by Pessan et al. [33] were considerably higher (65.7 and 30.1 ppm F^−^ at 1 and 12 h after brushing with 1000 ppm F^−^) compared to those found in the present study for Elmex^®^ (21.80 and 11.32 ppm F-, respectively).

Differences in sampling protocols may help explain the discrepancy in absolute fluoride levels between studies. While Pessan et al. [33] collected biofilm from all buccal and lingual surfaces, the present study used site-specific sampling by quadrants and dental arch. Solid biofilm was collected from upper molars, due to greater mass accumulation allowing its separation from fluid, while total biofilm was taken off from lower teeth, where separation was not feasible. These methodological differences may have influenced fluoride retention and should be considered in data interpretation. This interpretation is consistent with the high intraoral variability in fluoride levels previously discussed by Larsen et al. [36].

In contrast to our findings, in which salivary fluoride concentrations were higher than those in biofilm fluid, Larsen et al. [36] reported the opposite pattern, with biofilm fluid exhibiting approximately twice the fluoride concentration found in saliva. These differences may be related to methodological aspects, as Larsen et al. [36] analyzed naturally accumulated biofilm from multiple intraoral sites without prior control of fluoride exposure, whereas in our study, biofilm samples were site-specific and collected after controlled brushing periods.

The high fluoride concentration found in solid or total biofilm, compared to saliva and biofilm fluid, can be explained by two distinct retention mechanisms: (1) biological reservoirs, where small amounts of fluoride bind to intracellular and extracellular bacterial components via calcium bridges (Ca²⁺) at anionic sites on bacterial surfaces, a process influenced by pH and the availability of binding sites; and (2) mineral reservoirs, consisting of CaF₂-like deposits that precipitate when high calcium and fluoride concentrations coexist in the biofilm, independently of bacterial binding sites. The latter mechanism appears to account for most of the fluoride retained in the biofilm [39, 44, 45].

Considering the relevance of calcium, a limitation identified in the present study is the inability to evaluate calcium levels in the samples due to the insufficient amount of material available for analysis. Considering that calcium plays a fundamental role in fluoride retention, its quantification could provide additional information on the mechanisms involved in the interaction between ions in the biological matrix. It would be interesting, in future studies, to incorporate the Arsenazo III method for quantifying calcium concentrations in the collected samples, using a microplate reader, to enable the cross-referencing of data from these two components, which are highly relevant in this interaction, as performed by Kondo et al. [34]. Other important information that shall be analyzed in the future is the presence of Ti in saliva and biofilm by Atomic Absorbance Spectroscopy, especially because TiF₄ did not increase fluoride retention compared with AmF toothpaste, suggesting that titanium-related mechanisms should also be considered to explain its superiority to control tooth demineralization previously found in other studies [17, 22, 25].

Regarding the participants opinion, tooth staining was a complaint found in 50% of participants who used TiF_4_ toothpaste, 35% of those who used the TiF_4_/Ch toothpaste, and 15% of participants in the placebo group, whereas no staining complaints were reported for the commercial toothpaste. Tooth yellowing observed in this study also occurred with the placebo toothpaste. This effect may be attributed to the lower abrasivity of the experimental formulations compared to the commercial one (Elmex^®^), which may have been more effective in removing extrinsic stains. This assumption is supported by previous scanning electron microscopy (SEM) analysis of the toothpastes abrasive particles, which indicated low abrasive potential of the experimental formulations [30]. Francese et al. [30], who also used the same toothpastes in their study, did not report complaints, which may be attributed to the shorter experimental period (5 vs. 7 days). Staining may also have resulted from the interaction of titanium with the tooth surface, since tooth discoloration has been reported with the use of dentifrices containing metallic fluorides [46]. Nevertheless, with appropriate formulation adjustments, it may be possible to minimize and balance this disadvantage in the future. In addition, the TiF_4_/Ch toothpaste was the least pleasing in terms of flavor, which may negatively affect user acceptance and should be considered when evaluating its future clinical or commercial applicability, especially because chitosan did not provide an additional benefit in intraoral fluoride retention under the present conditions. Future toothpaste formulations shall also include different flavors in order to minimize this concern.

Although abrasivity was evaluated by Francese et al. [30], the method was semi-quantitative. Our protocol did not include relative dentin abrasivity (RDA) or relative enamel abrasivity (REA) values, which are internationally accepted measures for abrasivity (gold-standard). Other important point that may be contributed is that the 12 h biofilm accumulation adopted here may have intensified stain formation and detection. Nevertheless, the detected stains were easily removed by prophylaxis at the end of each phase, with no permanent effects. It would be of interest to perform reflectance spectrophotometry (tooth color measurement) comparing the experimental toothpastes and Elmex^®^.

Further studies are also needed to elucidate the influence of other components of the biofilm matrix on fluoride retention and release, as well as to assess the potential clinical impacts of these interactions on the control of dental caries.

## Conclusion

In conclusion, the experimental TiF₄ toothpastes, regardless of chitosan addition, maintained intraoral fluoride levels comparable to those of the commercial Elmex^®^ formulation. This effect was particularly evident 1 h after use in saliva and biofilm fluid, and up to 12 h in total and solid biofilm, where fluoride remains bound to biofilm components. The persistence of fluoride within the biofilm (both total and solid fractions) reinforces the relevance of this formulation, especially for individuals with increased susceptibility to dental caries. Nevertheless, further studies are required to elucidate the mechanisms underlying fluoride retention, as well as to investigate additional properties and the protective effects of these experimental toothpastes. Overall, given the absence of significant differences between the TiF₄ formulations and the commercial product, our findings suggest that these toothpastes are equivalent in their ability to maintain intraoral fluoride reservoirs, supporting further investigation of their potential in caries models.

## Data Availability

The datasets generated and/or analyzed during the current study are available from the corresponding author on reasonable request.
